# Marine Benthic Cyanobacteria as Potential Bioresources for Rare Earth Element Management: Insights from Lanthanum Bioaccumulation and Strain-Specific Physiological Responses

**DOI:** 10.3390/biology15171551

**Published:** 2026-09-04

**Authors:** Ekaterina Sergeevna Miroshnichenko, Anastasiia Andreevna Blaginina, Daria Sergeevna Balycheva, Vyacheslav Nikolaevich Lishaev, Sergey Victorovich Kapranov, Mikhail Vitalievich Simokon, Svetlana Nikolaevna Zheleznova, Vitaliy Ivanovich Ryabushko

**Affiliations:** 1 A.O. Kovalevsky Institute of Biology of the Southern Seas of RAS, Nakhimov Ave. 2, 299011 Sevastopol, Russia; esmiroshnichenko@ibss-ras.ru (E.S.M.); aablagini@ibss-ras.ru (A.A.B.); megarafik@gmail.com (V.N.L.); sergey.v.kapranov@yandex.ru (S.V.K.); zheleznovasveta@yandex.ru (S.N.Z.); rabushko2006@yandex.ru (V.I.R.); 2 Pacific Fisheries Research Center (TINRO-Center), Shevchenko Lane 4, 690091 Vladivostok, Russia; mikhail.simokon@tinro.vniro.ru

**Keywords:** Black Sea cyanobacteria, lanthanum hyperaccumulation, strain-specific responses, hormesis, intracellular inclusions, multi-metallic binding, bioremediation

## Abstract

Marine cyanobacteria represent a promising but underexplored bioresource for rare earth element (REE) management. This study investigated the effects of lanthanum (La, 10 mg L^−1^) on four cyanobacterial strains isolated from Black Sea periphyton: *Desertifilum tharense* AqMaPh-IBSS-CYA-1, *Toxifilum mysidocida* AqMaPh-IBSS-CYA-10, *Salileptolyngbya* sp. IBSS-CYA-8, and *Cyanobium* sp. AqMaPh-IBSS-CYA-15. La induced a delayed hormetic response in *Cyanobium* sp. Under suboptimal light and temperature conditions, La supplementation supported 100% survival of all strains over 35 days, whereas controls perished. SEM-EDX indicated predominantly intracellular La localization, with surface-bound La detected only sporadically. ICP-MS revealed hyperaccumulation of La (9.5–13.3 mg g^−1^ dry weight, 110–226-fold enrichment) and co-accumulation of light REEs in filamentous strains. Strain-specific responses suggest distinct protection mechanisms: *T. mysidocida* may employ metal efflux and polyphosphate granule sequestration, suggesting potential for REE recovery; *Salileptolyngbya* sp. appears to show broad-spectrum metal binding, potentially useful for bioremediation; *D. tharense* seems to be the most sensitive under the tested conditions, suggesting potential as a REE bioindicator. These findings are preliminary, but they suggest that La may act not only as a toxicant but also as a metabolic modulator, highlighting the potential of these cyanobacteria as bioresources for sustainable REE management.

## Simple Summary

Modern technologies rely on rare earth elements, but due to their mining and use, these metals are released into the environment, posing risks to marine life. This study exposed four marine cyanobacterial strains to lanthanum, one of the most abundant rare earth element found in nature, and showed how each strain reacted. Remarkably, one strain reached nearly twice the maximum cell density of the control after an initial shock, and all strains survived under stressful light and temperature conditions when lanthanum was present, whereas they perished without it. This study reveals that certain cyanobacteria can absorb large amounts of lanthanum, up to 226-fold higher than in the control, and stored it predominantly inside their cells. Different strains responded in unique ways: one bound metals in a thick slime layer, another locked them in internal granules, and a third showed high sensitivity to the metal. These findings suggest that specific cyanobacteria may be used to clean up polluted waters (bioremediation), take up valuable metals from waste (recovery), or even act as early warning systems for pollution (bioindication). This natural, low-cost approach could help reduce environmental damage and support sustainable technology by turning a pollution problem into a resource.

## 1. Introduction

Rare earth elements (REEs), including lanthanides (Ln), scandium (Sc), and yttrium (Y) [1], are critically important resources for modern high-tech industries such as optoelectronics, electric vehicle manufacturing, superconductors, and renewable energy [2]. Their widespread use is accompanied by an increased environmental burden, posing risks to ecosystems and human health [3,4]. Global REE reserves are estimated at about 90 million tons, of which 49% are concentrated in China, 24% in Brazil, 22% in India, Australia, Russia, and Vietnam [5].

REEs enter marine ecosystems from both anthropogenic and natural sources, including rock weathering, aeolian transport, and hydrothermal activity [6,7,8]. Lanthanum (La), cerium (Ce), and neodymium (Nd) are the most abundant REEs in the World Ocean, with concentrations decreasing from light to heavy elements, and higher levels in coastal and Arctic zones than in the open ocean [8]. For example, La concentrations ranges from 0.0036 to 0.0460 nmol∙L^−1^ in the Pacific [9], 0.0137 and 0.0818 nmol∙L^−1^ in Arctic estuaries [10], and 0.0140–0.1113 nmol∙L^−1^ in Tokyo Bay [11]. According to long–term monitoring data (1991–2007), REE concentrations in the Black Sea off the southern coast of Crimea ranged from 0.0008 nmol∙L^−1^ (samarium (Sm)) to 0.22 nmol∙L^−1^ (La) [12]. More recent data from the southern coast of Crimea and the Sevastopol area report concentrations from 0.0006–0.0049 nmol∙L^−1^ (for lutetium (Lu)) to 0.011–0.083 nmol∙L^−1^ (for Ce) and 0.008–0.108 nmol∙L^−1^ (for La), with scandium (Sc) measured at 1.15 nmol∙L^−1^ [13].

In the marine environment, the distribution, transport, and bioavailability of REEs are determined by complex biogeochemical cycles [6,14]. Transformations between dissolved and solid phases play a key role in these processes, as does the interaction with marine biota, particularly microorganisms. Among them, cyanobacteria have been shown to possess significant capacity for the REE binding. This has been demonstrated in model strains as well as freshwater and soil species, such as *Limnospira platensis* (Gomont) K.R.S.Santos & Hentschke and representatives of the genera *Anabaena* Bory ex Bornet & Flahault, *Nostoc* Vaucher ex Bornet & Flahault, and *Phormidium* Kützing ex Gomont [15,16,17,18,19,20,21,22,23]. However, interactions between marine, especially benthic, cyanobacteria and REEs have received limited attention, with only a few studies published, such as that on *Limnospira maxima* (Setchell & N.L.Gardner) Nowicka-Krawczyk, Mühlsteinová & Hauer [15].

Marine benthic cyanobacteria, which form biofilms and mats—multilayered, self-sustaining, and largely closed microbial communities—represent a remarkable reservoir of phylogenetic and functional diversity. These cyanobacteria have specialized adaptations and biochemical pathways that allow them to synthesize complex secondary metabolites and stress-responsive biocompounds [24,25,26]. Marine cyanobacteria have been of particular interest to biotechnological studies [27,28,29]. The exceptional resistance of biofilm-based communities to diverse environmental stressors, along with their specific responses to changes in nutrients and toxicants levels in the water [25,30,31], suggests that they are ideal model organisms for pioneering research into their interactions with REEs.

According to recent studies [32], REEs are not essential elements for most organisms in the traditional sense. Their primarily biological effect is associated with their similarity in ionic radius and coordination number to certain ions, particularly calcium (Ca^2+^). This similarity results in competitive inhibition of Ca uptake and disruption of calcium metabolism, which is the main cause of REE toxicity in organisms [33,34]. The toxicity and risk associated with REEs are also influenced by their complexation with phosphates, which limits their bioavailability to phototrophic organisms [4,35]. Additionally, REEs may participate in certain physiological processes, such as the formation of reactive oxygen species [19]. The extent of toxicity varies among different REEs as well as among species. At low doses, REEs can have stimulatory or beneficial effects on metabolic processes [32]. This phenomenon is known as hormesis, a dose-dependent response in which low or sublethal levels of stressors enhance biological performance [36]. Understanding how organisms have adapted to adsorb, accumulate, and utilize REEs can inspire the development of novel technologies, chemical transformations, and enchanced REE processing and recycling methods [17,37,38,39,40].

In recent years, the uptake of metals by cyanobacterial biomass has garnered significant interest across various biotechnological applications [41,42], particularly those involving marine cyanobacteria [43]. The uptake of REEs by cyanobacteria can occur via binding on the cell surface (biosorption) or through uptake across cell membranes, where REE binding occurs intracellularly (bioaccumulation) [16,38,44,45]. Extracellular polymeric substances (EPS) are known to have a high binding affinity for metals, including REEs [16,45,46].

The findings from these studies have significant implications for developing sustainable and efficient methods of metal recovery and recycling. Traditional physicochemical methods for extracting REEs from wastewater are energy intensive, costly, and generate secondary pollution [38,47]. Currently, the accumulated knowledge about the effects of REEs on organisms has led to research into more advanced, environmentally friendly, and cost-effective technologies for the REEs recycling in industrial processes [14,17,38]. The high biosorption and bioaccumulation capacities of cyanobacteria, which can achieve up to 99% recovery of REEs from solutions within minutes to hours [15,17,18,20,22,48], along with their ability to form REE nanoparticles [18,49,50] make them particularly promising candidates for sustainable and economically viable alternatives to current technologies [14,17]. Additionally, the REE profile in cyanobacteria can serve as a valuable indicator for exploring marine ecological processes and can be used to monitor pollution sources [4,27]). Moreover, the specific responses of individual cyanobacterial strains to REEs can facilitate the development of biosensors for environmental monitoring [4,40,51,52].

To elucidate the poorly understood interactions between marine benthic cyanobacteria and rare earth elements, the objective of this exploratory study was to investigate the strain-specific bioaccumulation capacity, localization patterns, and physiological responses of four Black Sea periphytic isolates to lanthanum exposure. Using a multi-method approach (growth kinetics, phenotypic characterization, SEM-EDX, ICP-MS, and in silico proteomic analysis) we aimed to generate testable hypotheses about their protective mechanisms and to assess whether the observed responses could guide future biotechnological applications.

## 2. Materials and Methods

### 2.1. Isolation and Characterization of Cyanobacterial Strains

The study used four cyanobacterial strains isolated from the periphyton of the Black Sea in the Sevastopol area (44.61577° N, 33.50329° E). The strains *Desertifilum tharense* AqMaPh-IBSS-CYA-1, *Toxifilum mysidocida* AqMaPh-IBSS-CYA-10, *Salileptolyngbya* sp. IBSS-CYA-8 and *Cyanobium* sp. AqMaPh-IBSS-CYA-15 have been deposited in the Microalgal and Cyanobacterial Collection of the Department of Aquaculture and Marine Pharmacology at the A. O. Kovalevsky Institute of Biology of the Southern Seas of RAS (IBSS RAS).

Biomass was scraped from the substrates using a scalpel and transferred to flasks containing sterile BG-11 nutrient medium [53] prepared with natural seawater at a salinity of 18 psu, without the addition of trace elements (marine BG-11). Cultivation was carried out at 23 ± 2 °C under natural light (NL, north-facing windows, approximate irradiance 30 μmol photons∙m^−2^∙s^−1^) for two weeks. After signs of growth appeared in the mixed culture, single colonies were selected and transferred to Petri dishes containing 25 mL of fresh medium. Following two weeks of cultivation, the samples were purified under identical conditions by serial subculturing with Pasteur micropipettes to obtain axenic monocultures [53]. All manipulations were performed in sterile conditions using materials and instruments sterilized either in a dry heat sterilizer (GP-80 MO, Kasimov Instrument-Making Plant, Kasimov, Russia) at 180 °C for 1.5 h, or in a Tuttnauer 2540 ML autoclave (Tuttnauer, Tel Aviv, Israel) at 121 °C and 1 atm for 20 min. Culture purity was monitored using a Bresser BioTrino light microscope (Bresser Optics, Rhede, Germany). The isolated cultures were identified using a polyphasic approach, combining morphological characteristics according to specialized taxonomic guides [54,55,56,57] with phylogenetic analysis via PCR amplification and sequencing of the 16S rRNA gene (primers CYA106F and CYA781R) performed at the “Proteomics and Metagenomics” shared facilities center within IBSS RAS. Protocols and a detailed description of the technique are provided in Miroshnichenko et al. [26].

**Figure 1:**
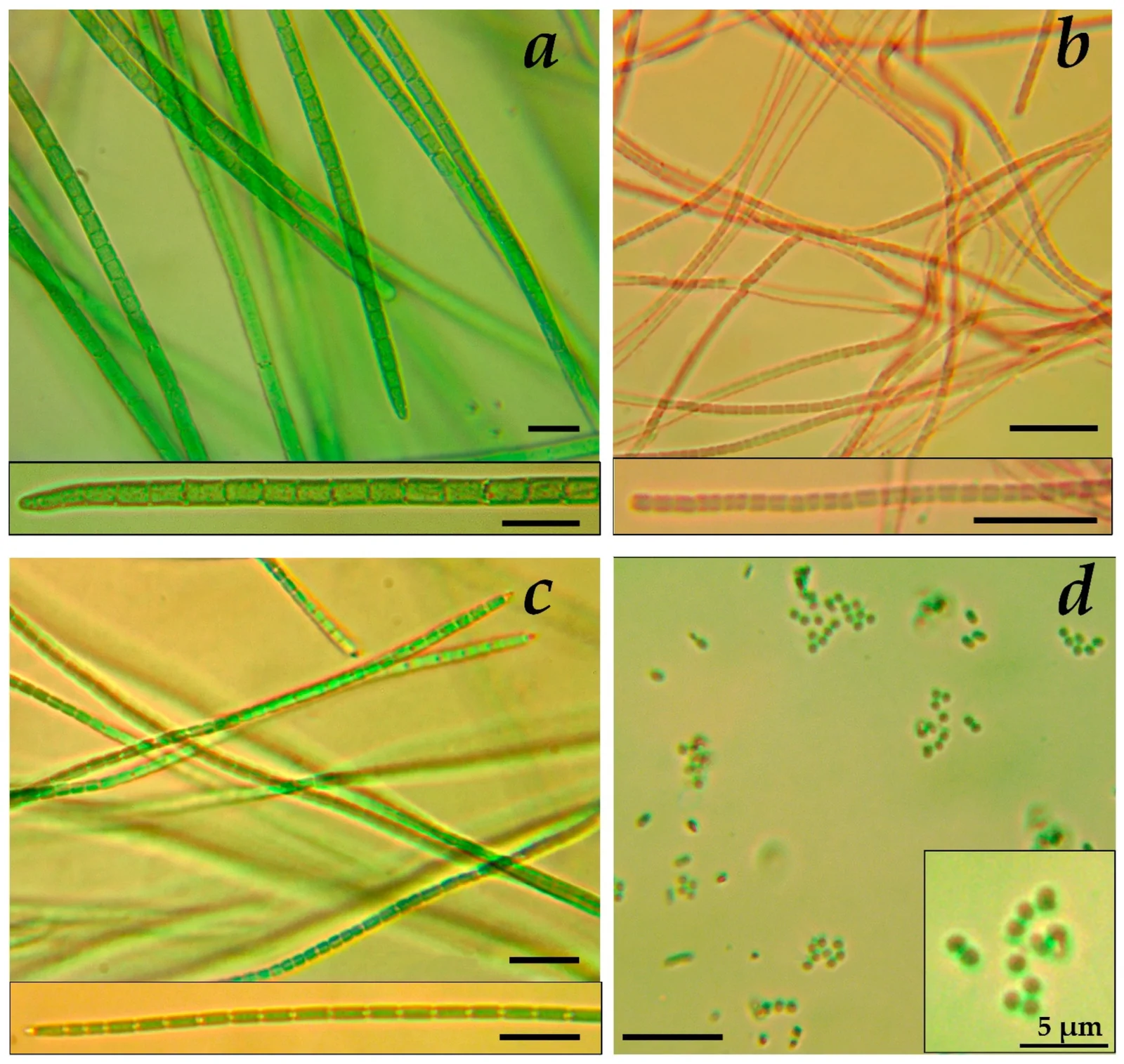
Light micrographs of isolated strains in light microscopy: (**a**)—*Desertifilum tharense* AqMaPh-IBSS-CYA-1; (**b**)—*Salileptolyngbya* sp. IBSS-CYA-8; (**c**)—*Toxifilum mysidocida* AqMaPh-IBSS-CYA-10; (**d**)—*Cyanobium* sp. AqMaPh-IBSS-CYA-15. Scale bar: 10 μm.

To study the bioaccumulation potential of lanthanum, four strains of cyanobacteria were used (Figure 1).

Strain *Desertifilum tharense* AqMaPh-IBSS-CYA-1

Based on phylogenetic analysis, this strain belongs to the filamentous cyanobacteria (Cyanophyceae) of the genus *Desertifilum* P.K. Dadheech & L. Krienitz, 2012, family Desertifilaceae Hasler, Casamatta, Dvorák & Poulícová 2017, and order Desertifilales Strunecký & Mareš 2023 [58]. It exhibits 99.84% sequence similarity to the type species (holotype) *Desertifilum tharense* Dadheech & Krienitz 2012 (strain PD2001/TDC17T GenBank) [54]. The morphological features of strain *D. tharense* CYA-1 are shown in Figure 1a. The thallus is thin, pale to bright blue-green. Filaments are solitary or densely entangled, of variable length, and exhibit motility (gliding and oscillating) and phototaxis. The trichomes are 3.0–4.2 µm wide. Trichomes are attenuated at the ends and slightly constricted at the cross-walls. Sheaths are thin, colourless, and agglutinated to the trichome. Cells are isodiametric to elongated, (2.3)3.2–4.3 µm in length, with homogeneous cell contents containing granules. Apical cells are long-conical with a rounded apex, but their shape can be variable; a calyptra is absent. Trichomes divide via intracellular breakage. Representatives of this species are commonly found in desert biological crusts, saline lakes, geothermal and mineral springs, as well as in polluted waters, indicating a broad geographical distribution [54,58,59,60,61].

Strain *Salileptolyngbya* sp. IBSS-CYA-8

Phylogenetic analysis classifies this strain within the genus *Salileptolyngbya* W.G. Zhou, 2018, family Nodosilineaceae Strunecký & Mareš 2023 and order Nodosilineales Strunecký & Mareš 2023 [58]. It shows 98.9–99.4% similarity to closely related *Salileptolyngbya* spp. strains from the LEGE culture collection [26]. The sequence similarity to the holotype *Salileptolyngbya diazotrophica* W.G. Zhou & J. Ling 2018 [56] is 96.0%, supporting its classification at the genus level. The morphological characteristics of Black Sea strain *Salileptolyngbya* sp. CYA-8 are presented in Figure 1b. The thallus is thin, ranging in color from pink to carmine-red or brownish-red. Filaments are straight or slightly curved, unbranched, and densely but unevenly intertwined, forming mats, films or parallel bundles. Sheaths are mostly confluent into an amorphous matrix, though filaments remain within firm sheaths. Trichomes are brownish to pale purple, 0.9–1.5 µm wide, constricted at the ungranulated cross-walls, not attenuated at the ends, and lack heterocysts. Cells are cylindrical, slightly longer than wide (0.9–2.3 µm in length), with homogeneous content lacking aerotopes but with distinct chromatoplasma. The apical cell is elongated and rounded, without a calyptra. Reproduction occurs via hormogonia. This genus is typical of saline environments.

Strain *Toxifilum mysidocida* AqMaPh-IBSS-CYA-10

Phylogenetic analysis identifies this strain as belonging to the genus *Toxifilum* Zimba, Huang, Foley & Linton, 2017, family Oculatellaceae Mai & J.R. Johansen 2018 and order Oculatellales Strunecký & Mareš 2023 [58], showing 99.56% similarity with the type species *T. mysidocida* Zimba, I.S. Huang, Foley & Linton 2017 [57]. The morphology of *T. mysidocida* CYA-10 is shown in Figure 1c. The thallus of the strain is dark green, dense, forming a film or mat. Filaments are straight, curved, or intertwined, narrowing at the ends, slightly constricted at the cross-walls, unbranched, and exhibit gliding motility. Sheaths are thin, colorless, tightly attached to the trichome, and often barely visible under light microscopy. Trichomes are uniseriate and 1.3–1.7 µm wide. The apical end of the terminal cell is typically rounded, enclosed by a sheath. Cells are cylindrical, bright green, (1.4)2.2–4.8 µm in length. The cell content is differentiated into chromato- and centroplasm. Bright, refractive intracellular bodies and granules are located near the cell cross-walls. Plasmodesmata are present between cells. Reproduction occurs via necridic cells (necridia). It is a toxic, benthic marine species.

Strain *Cyanobium* sp. AqMaPh-IBSS-CYA-15

Phylogenetic analysis assigns this strain to the genus *Cyanobium* R. Rippka & G. Cohen-Bazire, 1983, family Prochlorococcaceae Komárek & Strunecký 2020, and order Synechococcales Hoffmann, Komárek & Kaštovský 2005 [58]. It shows 99.91% similarity to strain *Cyanobium* sp. LEGE 07175. The morphology of *Cyanobium* sp. CYA-15 is shown in Figure 1d. The strain is characterized by small, blue-green cells that are solitary or in pairs after division and do not form colonies. Cells are oval to ellipsoid, sometimes short rod-shaped, without gelatinous envelopes, and measure approximately 1–1.6 µm in length and 0.7(–1.2) µm in width. The chromatoplasm is usually well-defined. Cell division occurs via binary fission, with cells regaining their original shape and size before the next division. Members of this genus are obligately photoautotrophic [55,62].

### 2.2. Cultivation of Cyanobacteria in Lanthanum-Supplemented Medium

The experimental design consisted of four parts: (1) analysis of the growth kinetics of the unicellular *Cyanobium* sp. CYA-15 strain in the presence of 10 mg∙L^−1^ lanthanum over 7 days; (2) phenotypic analysis of the effect of 10 mg∙L^−1^ lanthanum on all four strains under suboptimal conditions over 35 days; (3) determination of the localization of lanthanum in filamentous strains after 14 days of exposure using SEM-EDX analysis; and (4) investigation of the long-term (30-day) effect of 10 mg∙L^−1^ lanthanum on the elemental composition of the biomass of filamentous strains.

In all experiments, marine BG-11 medium was used. A 100 mL lanthanum stock solution (1 g/L) was prepared using distilled water, supplemented with 0.312 g of lanthanum (III) nitrate hexahydrate (La(NO_3_)_3_·6H_2_O), (Len-reaktiv, St. Petersburg, Russia) and 1.44 mL of 0.5 M Na_2_H_2_EDTA∙2H_2_O (Len-reaktiv, St. Petersburg, Russia). The solution was then pasteurized. Subsequently, 10 mL of this solution was added to 990 mL of sterile marine BG-11. The final working medium contained 10 mg L^−1^ La and 74.7 µM total EDTA (2.7 µM from the BG-11 formula and 72 µM from the La stock). The experiments (parts 1–3) were conducted in three biological replicates.

#### 2.2.1. Analysis of Growth Kinetics of *Cyanobium* sp. CYA-15

To study the effect of lanthanum on cyanobacterial growth kinetics, the *Cyanobium* sp. CYA-15 strain was selected, owing to its rapid growth and unicellular morphology, which make it well suited for quantitative analyses. To evaluate the effect of 10 mg∙L^−1^ lanthanum on the growth kinetics, tubes containing 9 mL of sterile marine BG-11 medium supplemented with 10 mg∙L^−1^ lanthanum were inoculated with 1 mL of culture at an initial density of approximately 10^5^ cells ∙mL^−1^. The cultures were incubated for seven days at 23 ± 2 °C under natural light (NL). To estimate cyanobacterial abundance, cells were counted according to the method described in [63] using a hemocytometer (Goryaev’s chamber, LLC. Minimed, Bryansk, Russia) with a volume of 0.9 mm^3^ under a light microscope at 10 × 40 and 10 × 100 magnifications. Counts were performed on days 0, 1, 4 and 7 in triplicate for each sample, and the results were converted to cells per milliliter (cells∙mL^−1^).

#### 2.2.2. Phenotypic Analysis Under Suboptimal Conditions

One milliliter of inoculum from each strain with an initial density of approximately 10^5^ cells∙mL^−1^ was transferred into Petri dishes containing marine BG-11 medium supplemented with 10 mg∙L^−1^ La (experimental group), and into dishes without lanthanum (control group). To test the hypothesis of a potential stimulating effect of lanthanum, the experimental and corresponding control groups were cultivated under suboptimal conditions: 17 ± 2 °C and shaded natural lighting (SNL, located in the far corner of the laboratory, approximately 5 μmol photons∙m^−2^∙s^−1^). Simultaneously, an optimal control was maintained (23 ± 2 °C, NL). The condition of the cultures was assessed visually based on cultural characteristics and morphology under a light microscope (10 × 20, 10 × 40, 10 × 100 magnifications) on days 7, 14, 21, and 35.

#### 2.2.3. Determination of the Localization of Accumulated Lanthanum in Filamentous Cyanobacteria Using SEM-EDX Analysis

**Figure 2:**
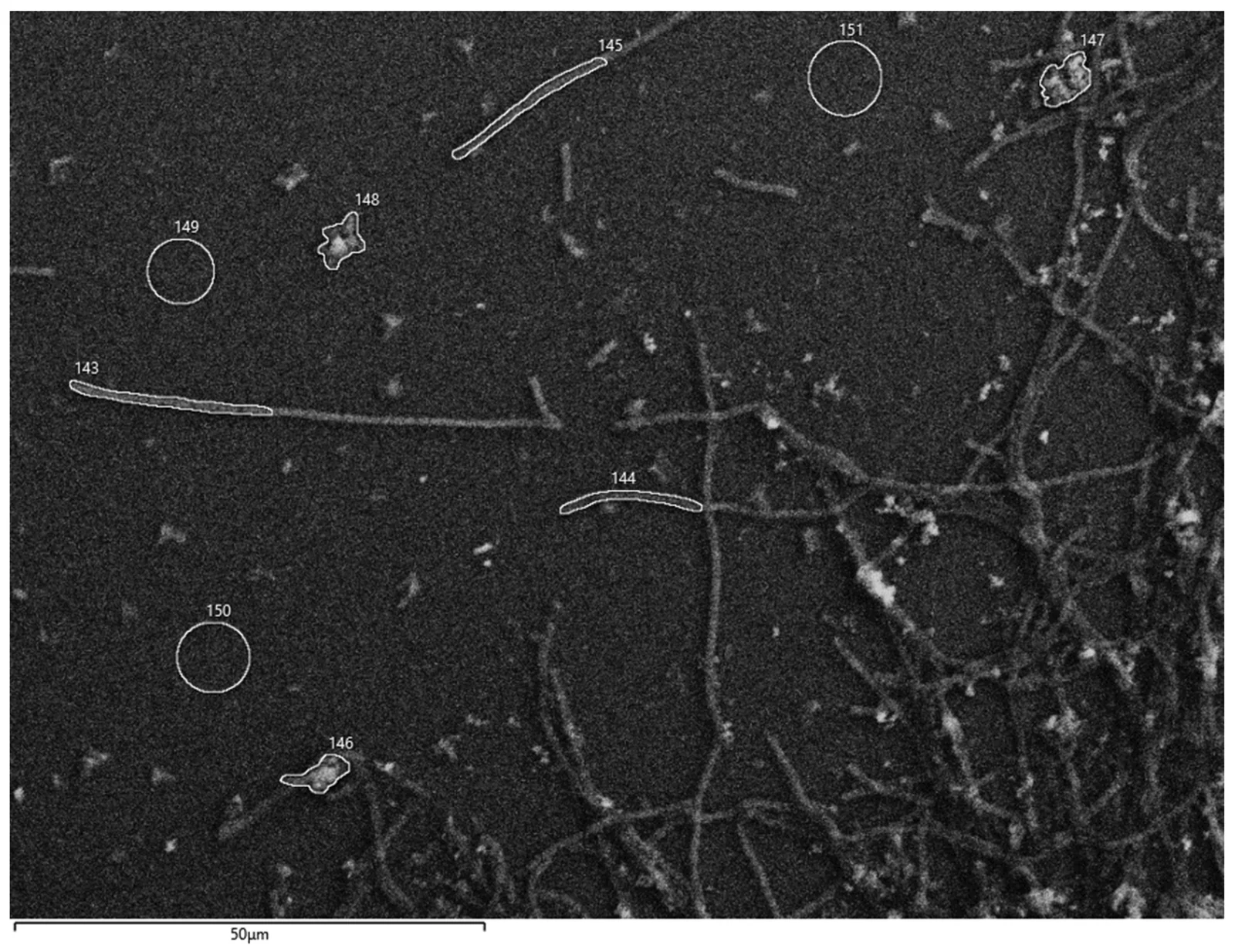
An example of field-of-view analysis: Spectra No. 143–145—intact filaments, Spectra No. 146–148—destroyed filaments, Spectra No. 149–151—background areas.

After 14 days of incubation of *Salileptolyngbya* sp. CYA-8, *D. tharense* CYA-1, and *T. mysidocida* CYA-10 strains in marine BG-11 medium supplemented with 10 mg∙L^−1^ La, a portion of the biomass was mechanically homogenized using a mortar and pestle. A drop of the crushed biomass, without prior EDTA washing, was applied as a thin layer to clean degreased coverslips. Samples were fixed in 1.25% glutaraldehyde at 6 °C overnight, followed by dehydration through a graded ethanol series (25%, 50%, 75%, and 96%) for 10 min at each concentration [64]. Subsequently, the samples were dried using a critical-point dryer (EM CPD300, Leica, Wetzlar, Germany) with CO_2_. EDX analysis was used to determine the chemical composition of the filaments and biomass surfaces [65]. To ensure electrical conductivity, the sample surfaces were sputter-coated with a thin layer of a gold-palladium alloy using an ion sputter-coater (ACE200, Leica, Wetzlar, Germany) prior to energy-dispersive X-ray spectroscopy (EDX) analysis with a Hitachi SU3500 scanning electron microscope (SEM) (Hitachi Ltd., Tokyo, Japan). EDX spectra were acquired using an Oxford Instruments Ultim Max detector (Abingdon, UK) integrated into the SEM. Ten fields-of-view were analyzed for each strain, each containing three intact filaments, three clusters of destroyed filaments, and three background areas (substrate without biomass). An example of the visual field analysis is shown in Figure 2.

For each measurement, an individual EDX spectrum was recorded. Elemental compositions were expressed as weight percentages (wt%). To eliminate the contribution of the gold-palladium coating applied during SEM sample preparation, the Au/Pd signals were subtracted from the EDX spectra using the instrument software. Because the interaction volume in SEM-EDX extends to a depth of several micrometers, the measured elemental composition represents the near-surface region of the cyanobacterial filaments or biomass rather than only the outermost surface.

#### 2.2.4. Cultivation in Lanthanum-Supplemented Medium and Biomass Elemental Analysis

Strains of filamentous cyanobacteria—*Salileptolyngbya* sp. CYA-8, *D. tharense* CYA-1, and *T. mysidocida* CYA-10—were cultivated for 30 days in marine BG-11 medium, both with 10 mg∙L^−1^ La supplementation and without lanthanum (control, C) under optimal conditions (23 ± 2 °C and NL). To determine the elemental composition, the biomass was collected by centrifugation, washed twice with isotonic sodium chloride solution (9 g∙L^−1^) and dried to constant weight in an RA-50/500 VZ drying oven (Micron, Shatura, Russia) at 105 °C for 24 h. The dry biomass was weighed using a Scout SPX222 analytical balance (OHAUS Ltd., Parsippany, NJ, USA). Approximately 0.2 g dry weight (DW) of each sample was then subjected to microwave-assisted acid digestion (Milestone Ethos EZ, Sorisole, Italy) in a mixture of high-purity nitric acid (3 mL) and hydrogen peroxide (0.5 mL, 30%). Element concentrations were determined on an Agilent 7700x inductively coupled plasma mass spectrometer (ICP-MS) (Santa-Clara, CA, USA) [66,67]. Due to the limited amount of biomass obtained from the 30-day cultivation experiment, the elemental analysis was performed on a single sample per treatment (control and La-supplemented) for each strain. Thus, the reported concentrations represent single measurements without technical or biological replicates and should be interpreted as preliminary estimates. Calibration solutions were prepared from multielement standard solutions IV-ICPMS-71A–D (Inorganic Ventures, Christiansburg, VA, USA) with certified element concentrations (10 mg∙L^−1^). An indium solution (200 μg∙mL^−1^) was used as the internal standard. The accuracy and reproducibility of the analysis (quality control) were ensured by using the certified reference material SRM^®^ 2976 “Mussel Tissue” (National Institute of Standards and Technology, Gaithersburg, MD, USA). The quantification uncertainty did not exceed 10%, and the recovery ranged from 85 to 123%. Detected element concentrations are expressed in μg∙g^−1^ DW.

### 2.3. In Silico Analysis of Homologous Proteomes

To generate hypotheses regarding metabolic shifts and physiological responses to lanthanum exposure, we performed an in silico analysis using publicly available proteomes of the closest phylogenetic relatives for each filamentous cyanobacterial strain. Reference proteomes were retrieved from the NCBI database [68] and UniProt [69] databases:

- for *Desertifilum tharense* CYA-1, the proteome of *Desertifilum tharense* IPPAS B-1220 (UniProt: UP000095472; taxid: 1781255 [70]) was used as its 16S rRNA gene sequence is identical (100% identity);
- for *Toxifilum mysidocida* CYA-10, the proteome of *Leptolyngbya* sp. CCY15150 (UniParc query: Leptolyngbya+sp+CCY15150&facets=database_facet%3A2500; taxid: 2767772 [71]) was selected based on its high 16S rRNA gene sequence identity (99.71%) to *Toxifilum mysidocida* MYSIDO1T (GenBank accession: KX375342);
- for *Salileptolyngbya* sp. CYA-8, the proteome of *Leptolyngbya iicbica* LK (UniProt: UP000292459; taxid 2294035 [72]), which shares 97.68% 16S rRNA gene sequence identity, was used.

The presence of genes encoding proteins and enzymes was assessed using keyword searches and BLASTp (https://blast.ncbi.nlm.nih.gov/Blast.cgi, web interface, accessed on 10 April 2026) [68] against the reference proteomes. Given the varying degrees of sequence similarity between the reference proteomes and the studied strains (ranging from 97.68% to 100%), all inferred functional assignments are treated as working hypotheses requiring direct experimental confirmation.

### 2.4. Statistical Analysis

Statistical analyses were conducted using PAST 4.17 [73]. Prior to the analysis, the Shapiro–Wilk and Levene’s tests were performed to verify the assumptions of normality and homogeneity of variances. Growth kinetics data were analyzed using two-way PERMANOVA (permutational multivariate analysis of variance) with the factors “treatment” (control, “C”, vs. experiment, “+La”) and “time” (days), based on Euclidean distance matrices with 9999 permutations. For elemental composition data derived from SEM-EDX, differences among groups (“background”, “intact filaments”, “destroyed filaments”) were assessed using two-way PERMANOVA, followed by pairwise one-way PERMANOVA for comparisons between intact and destroyed filaments within each strain, as well as among strains. To identify the elements contributing most to the observed dissimilarities, similarity percentage analysis (SIMPER) was performed using the Bray–Curtis similarity measure. For the ICP-MS elemental composition data (Table S2), no statistical tests were performed because the measurements were carried out in a single replicate. The fold-changes (experimental/control) are provided as descriptive indicators of the observed trends. All interpretations based on these data are presented as preliminary hypotheses requiring independent verification. Cell count results for *Cyanobium* sp. CYA-15 are presented as the mean ± standard deviation (SD), while SEM-EDX results are presented as medians and (25th–75th percentiles). A *p*-value below 0.05 was considered statistically significant. OriginPro 2024 software [74] was used to create the plots.

## 3. Results

### 3.1. Effect of Lanthanum on the Growth Kinetics of Cyanobium *sp.* CYA-15

**Figure 3:**
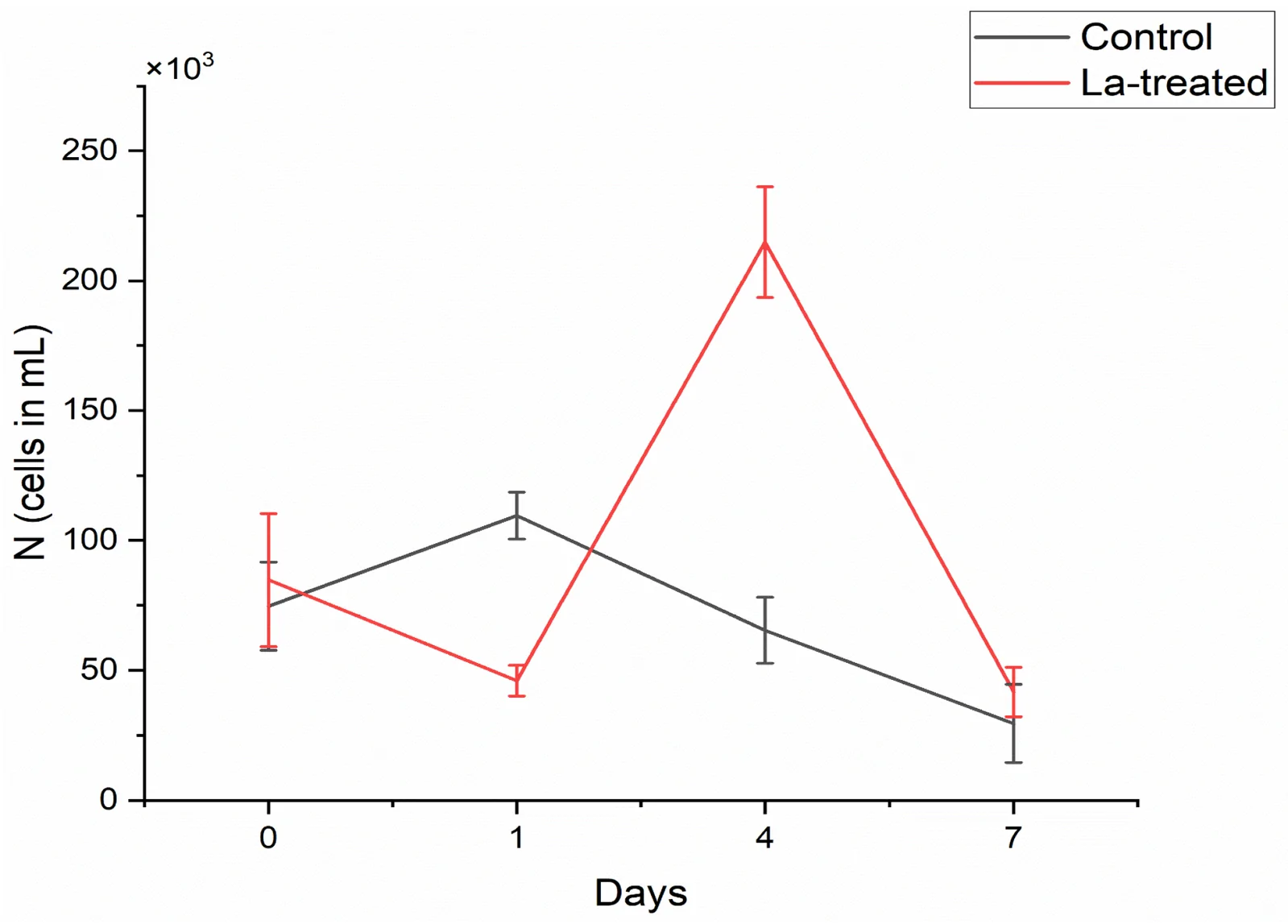
Growth kinetics of *Cyanobium* sp. CYA-15 during cultivation in marine BG-11 medium with the addition of 10 mg∙L^−1^ La compared to the control.

The growth kinetics of the unicellular cyanobacterium *Cyanobium* sp. CYA-15 in the presence of 10 mg∙L^−1^ La differed significantly from those of the control (Figure 3).

In the lanthanum-treated culture, the cell density decreased to 46.06 ± 5.87 thousand cells∙mL^−1^ on day 1. Over the following days, the cell density increased sharply, reaching a maximum on day 4. The maximum cell density in the La-treated group (214.91 ± 21.28 thousand cells ∙mL^−1^) was 1.96 times higher than the maximum value in the control group (109.67 ± 9.05 thousand cells ∙mL^−1^). In contrast, the control culture reached its maximum on day 1 and subsequently entered the decline phase, with the cell density decreasing to 29.60 ± 15.04 thousand cells ∙mL^−1^ by day 7. By day 7, the La-treated culture had also entered the decline phase, and its cell density had decreased to a level comparable to that of the control (41.64 ± 9.48 thousand cells ∙mL^−1^). Statistical analysis revealed a significant effect of lanthanum treatment on the growth dynamics of *Cyanobium* sp. CYA-15 (two-way PERMANOVA: F = 5.52, *p* = 0.0156).

### 3.2. Effect of Lanthanum on Cyanobacterial Strain Survival and Morphology Under Suboptimal Conditions

Cultivation under suboptimal light and temperature conditions resulted in the death of control cultures of *D. tharense* CYA-1 and *T. mysidocida* CYA-10 by day 35, whereas *Salileptolyngbya* sp. CYA-8 and *Cyanobium* sp. CYA-15 died by day 14. However, under the same stressful conditions but in a medium supplemented with 10 mg∙L^−1^ La, all four strains remained viable throughout the 35-day experiment.

**Figure 4:**
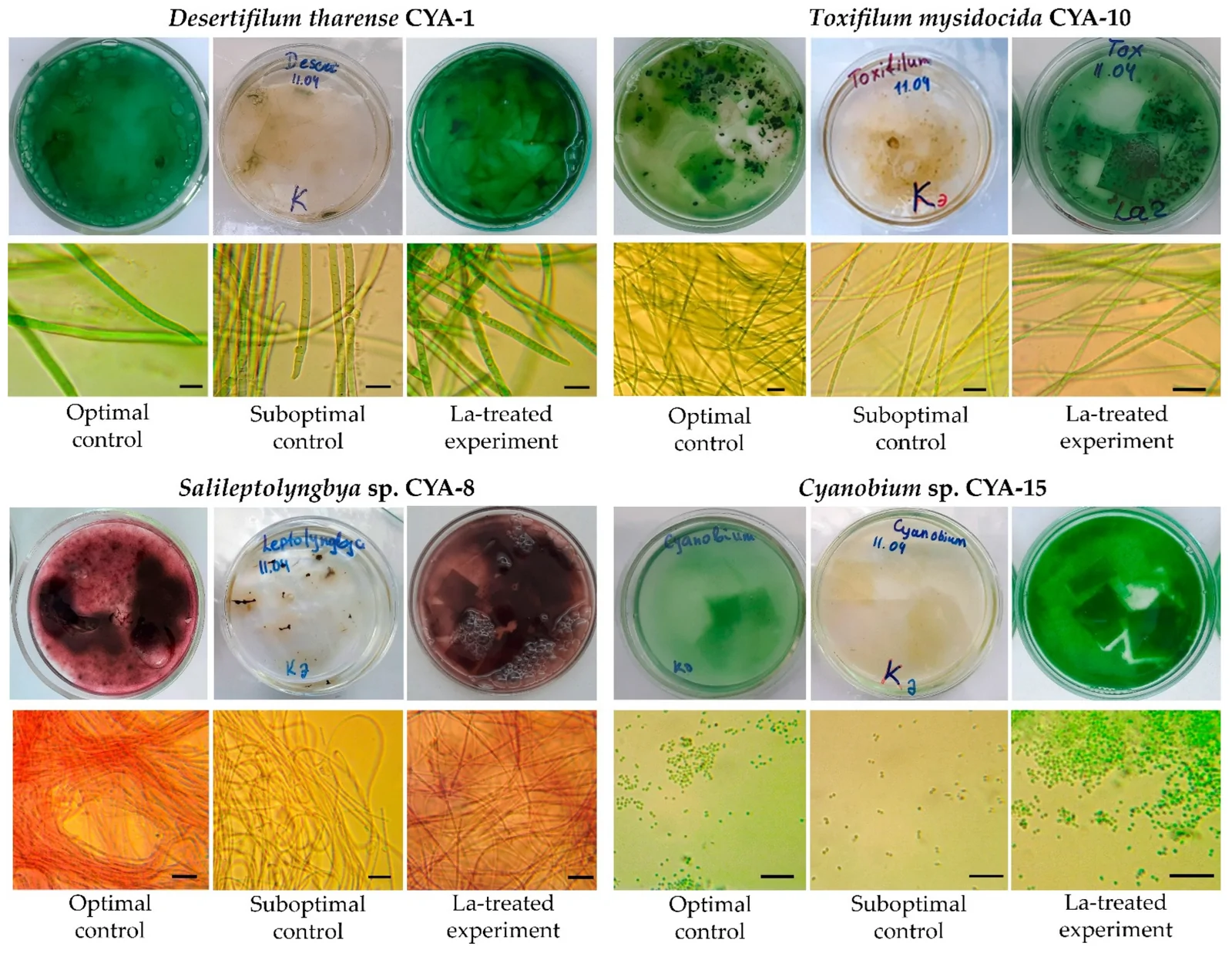
Phenotypic characteristics and morphology of cyanobacterial cultures at the end of the incubation period under experimental and control conditions. Scale bar = 10 μm.

Based on phenotypic and morphological characteristics (Figure 4), the experimental cultures under stressful conditions exhibited more pronounced growth and better preservation of cellular morphology then both the suboptimal control and the optimal control. The cells retained bright pigmentation, well-defined contours, and their characteristic size, with no signs of lysis, whereas degradation of cellular structures was observed in the suboptimal control.

**Figure 5:**
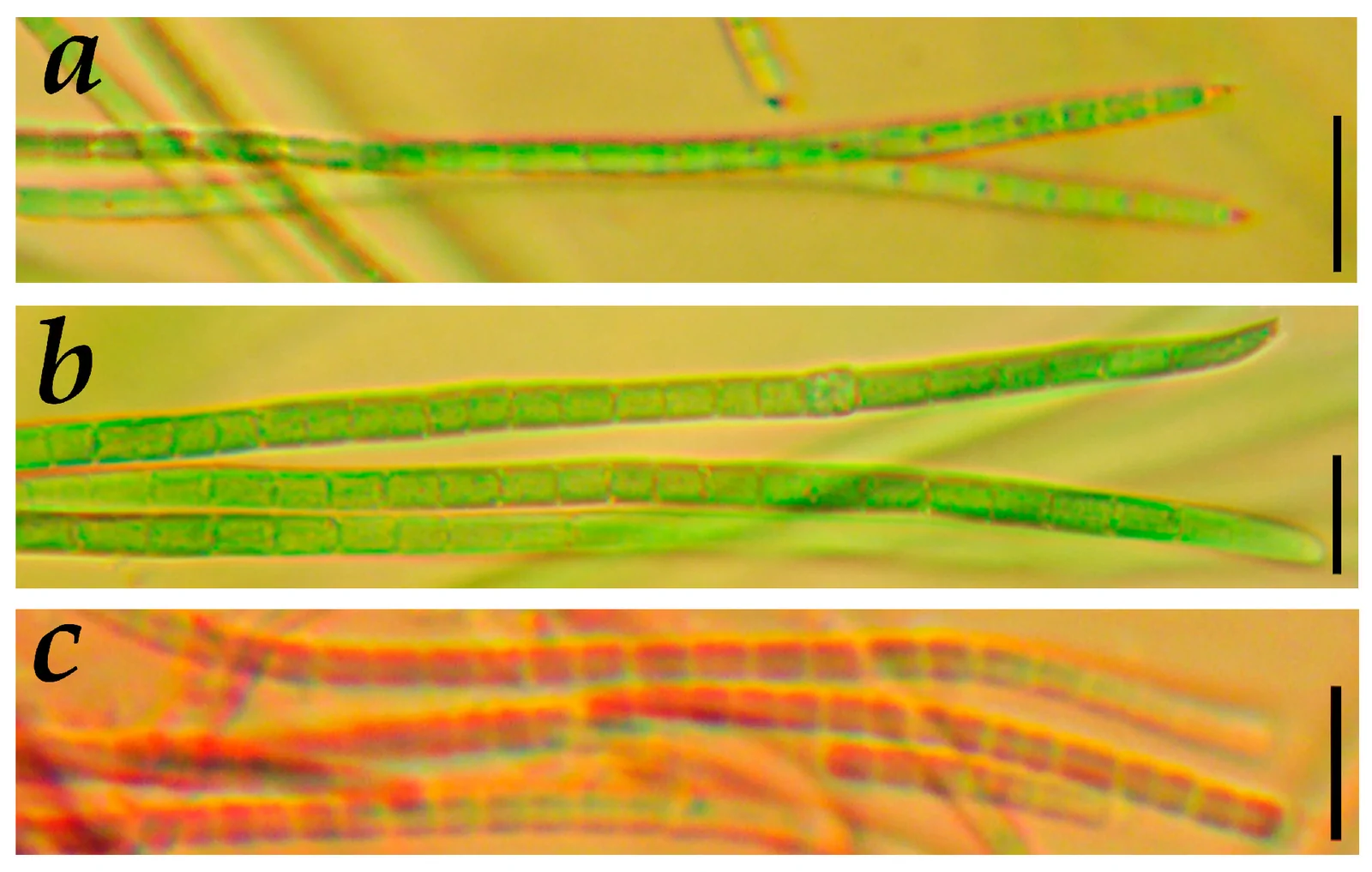
Light micrographs showing intracellular granules in cyanobacterial cultivated in La-supplemented medium: (**a**)—*T. mysidocida* CYA-10; (**b**)—*D. tharense* CYA-1; and (**c**)—*Salileptolyngbya* sp. CYA-8. Scale bar = 10 μm.

Light microscopy of the three filamentous strains after cultivation in La-supplemented medium revealed striking differences in intracellular granular structures (Figure 5).

*T. mysidocida* CYA-10 displayed numerous distinct, highly refractive polyphosphate granules. In contrast, *D. tharense* CYA-1 showed only fine granulation, whereas *Salileptolyngbya* sp. CYA-8 exhibited a homogeneous cytoplasm without visible intracellular granules.

### 3.3. Bioaccumulation of Lanthanum, Its Localization, and Changes in the Elemental Composition of Biomass

SEM-EDX analysis of intact filaments, destroyed biomass, and background in homogenized samples of *Salileptolyngbya* sp. CYA-8, *D. tharense* CYA-1 and *T. mysidocida* CYA-10 strains (Table S1) allowed for the assessment of La localization.

Background samples contained O and Si, as well as C, Na, Ca, and Mg, reflecting the elemental composition of the glass substrate. In contrast, N, P, S, Cl, and La were not detected. The multivariate elemental composition (C, N, Na, Mg, Ca, P, S, Cl) significantly differed among the “background”, “intact filaments” and “destroyed filaments” groups in all three strains (two-way PERMANOVA, *p* < 0.05). Disruption of the filaments resulted in the release of intracellular contents, leading to a marked increase in nutrient concentrations within the EDX interaction volume. Pairwise comparisons between intact and destroyed filaments for each strain also revealed highly significant differences in elemental composition (one-way PERMANOVA, *p* < 0.0001).

SIMPER analysis based on Bray-Curtis dissimilarities identified C as the primary element distinguishing intact from destroyed filaments in all strains (60.9–66.2% contribution). Cell destruction was accompanied by a marked increase in C, N, and P, while Na and Ca levels decreased. SIMPER analysis of intact filaments identified carbon (C) as the principal contributor to the observed differences (70.6%). The median C content (wt%) increased in the order: *Salileptolyngbya* sp. (13.2) < *T. mysidocida* (16.1) < *D. tharense* (17.5). N, Na, and Ca also contributed (8.5–9.9% each). Following cell disruption, C remained the dominant contributor (61.8%), but the N contribution increased (10.7%), and P contribution rose to 2.4%, consistent with the release of intracellular contents. The greatest relative increases in C (1.7-fold), and N (3.1-fold), as well as the largest increases in P and S following cell disruption, were observed in *Salileptolyngbya* sp. Pairwise one-way PERMANOVA of the elemental composition of intact filaments across strains revealed significant differences between *T. mysidocida* and *Salileptolyngbya* sp. (*p* = 0.040), as well as between *T. mysidocida* and *D. tharense* (*p* = 0.015). No significant differences were detected between *Salileptolyngbya* sp. and *D. tharense* (*p* = 0.42). The detection of intracellular nutrients, together with their marked increase after mechanical disruption, confirms that the analyzed material represented cyanobacterial biomass rather than the underlying substrate.

**Figure 6:**
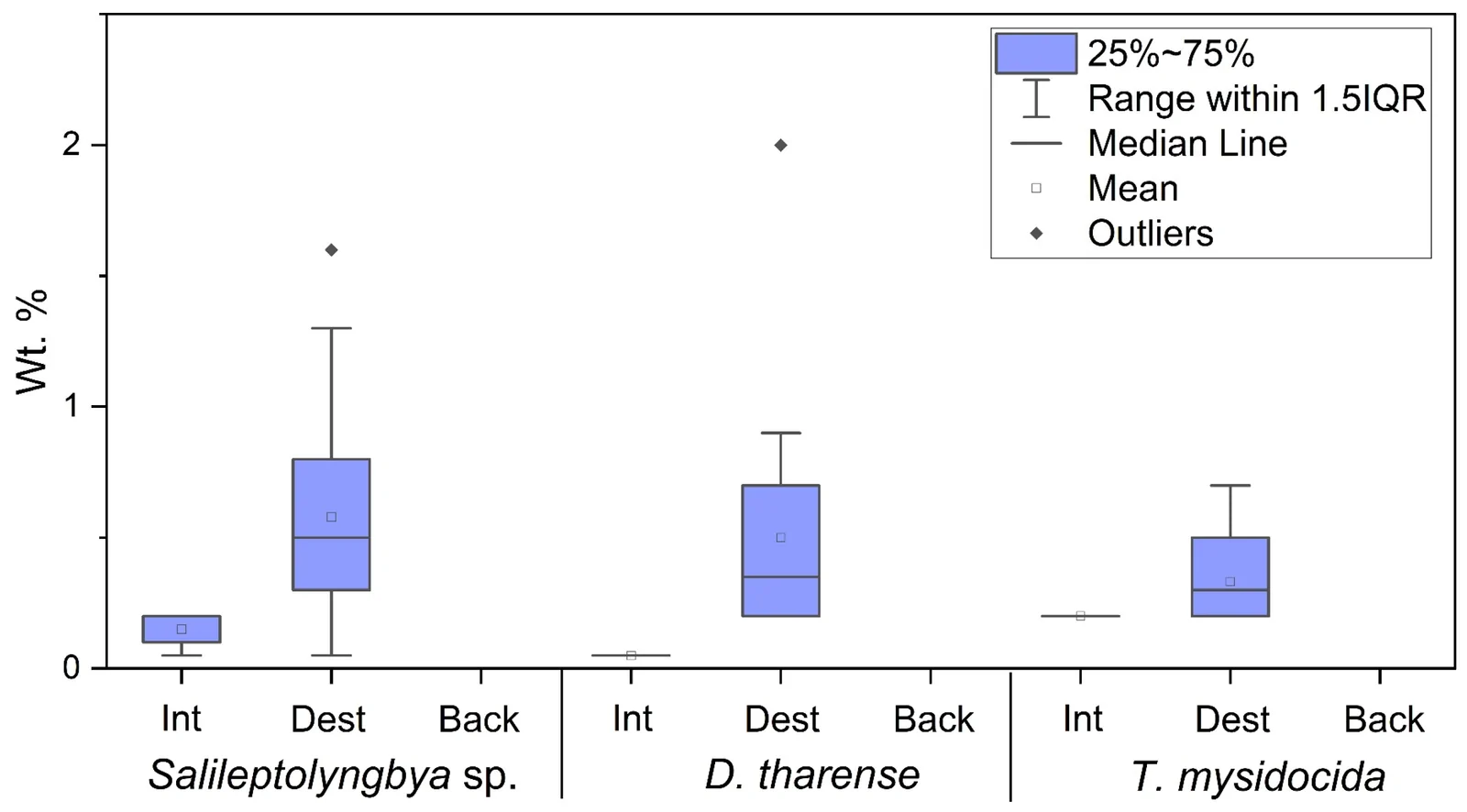
Lanthanum content in intact filaments (Int), destroyed filaments (Dest), and background (Back) samples of three cyanobacterial strains after incubation in La-supplemented medium.

Lanthanum was detected in both intact and disrupted filament samples of all three cyanobacterial strains but was not detected in the corresponding background samples, indicating its association with the biological material (Figure 6).

La was rarely detected on intact filament surfaces (0.05–0.2 wt%, single measurements), but its content increased sharply in destroyed biomass (0.3–0.5 wt%) in all strains (PERMANOVA, F = 61.83, *p* < 0.0001), whereas differences among strains were not significant (F = 1.52, *p* = 0.22), indicating predominantly intracellular localization. Notably, La was detected more frequently on intact filaments of *Salileptolyngbya* sp. (*n* = 5) compared to *T. mysidocida* (*n* = 1) and *D. tharense* (*n* = 1).

### 3.4. Long-Term Experiment and Elemental Analysis

Elemental analysis of the biomass of the three filamentous cyanobacterial strains by ICP-MS, after 30 days of cultivation in La-supplemented medium, revealed a high capacity for lanthanum accumulation, together with the accumulation of several other REEs (Table S2).

The La concentration in the biomass of the La-treated cultures increased by 110- to 226-fold relative to the control, reaching 9.5–13.3 mg∙g^−1^ dry weight (Table S2). The highest absolute La concentration was observed in *D. tharense*, whereas the greatest relative enrichment occurred in *Salileptolyngbya* sp. Concomitant accumulation of other light REEs (Ce—Gd) was observed in all strains. The apparent more-than-100-fold accumulation of Gd is difficult to explain consistently and was likely caused by mass-spectral interference from the hydrated lanthanum ions ^139^La(H_2_O)^2+^ with the ^157^Gd^2+^ signal. Accumulation of heavy REEs (Dy—Lu) was insignificant or remained at the control level.

Lanthanum exposure was accompanied by changes in the elemental composition of biomass in all three cyanobacterial strains, with apparent strain-specific patterns. Among the macronutrients (Table S2), consistent responses were observed in all strains: a decrease in calcium (Ca), an increase in magnesium (Mg) (highest in *D. tharense*), and an increase in sodium (Na) and potassium (K) in *Salileptolyngbya* sp. and *T. mysidocida* against a decrease in these elements in *D. tharense*. Phosphorus (P) decreased in *T. mysidocida* and *D. tharense*, while sulfur (S) increased exclusively in *Salileptolyngbya* sp.

For trace elements (Table S2), there was a notable increase in selenium concentrations in all three strains, ranging from 200- to 485-fold. Although the magnitude of the apparent enrichment of Se may have been affected by interferences involving La(H_2_O)^2+^ and, to a lesser extent, Gd^2+^, the overall increase in the Se signal was observed consistently in all strains. Iron (Fe) content decreased in all strains, while copper (Cu) and zinc (Zn) increased in *Salileptolyngbya* sp. and *D. tharense*, but decreased in *T. mysidocida*. Manganese (Mn) declined in *T. mysidocida*, while it remained stable in the other two strains. Molybdenum (Mo) increased in *T. mysidocida* and *D. tharense*, but decreased in *Salileptolyngbya* sp., whereas nickel (Ni) increased sharply (37-fold) in the latter strain only. Cobalt (Co) showed a slight decrease across strains, and vanadium (V) increased only in *D. tharense*. Notably, the control *Salileptolyngbya* sp. strain had unusually high boron (B) content.

The heavy metal dynamics (Table S2) were also strain-specific. *T. mysidocida* showed a decrease in Cr, Sr, Ag, Ba, and Pb, whereas *Salileptolyngbya* sp. showed an increase in Cr, Sr, Ba, and Pb, but a decrease in Ag and Cd. *D. tharense* showed an increase in Cr, Ag, and Ba, alongside a decrease in Sr, Cd, and Pb.

## 4. Discussion

Cyanobacteria have served as model organisms for investigating the toxic effects of metals for decades [75,76]. These studies laid the foundation for understanding dose-dependent effects and stimulated the search for strains with unique adaptive capabilities. This study demonstrates that lanthanum exerts complex, strain-specific effects on marine benthic cyanobacteria, extending beyond simple toxicity. At the tested concentration (10 mg∙L^−1^), La acted as a stressor that modulated growth dynamics, enhanced tolerance to stress conditions, and induced substantial hyperaccumulation of La and co-accumulation of other elements. However, the observed responses must be interpreted with caution, as the experimental concentration is significantly higher than typical environmental levels (0.15 ng∙L^−1^ in the Black Sea [13], and the biological significance under environmentally relevant conditions remains to be established.

### 4.1. Stress Adaptation and Growth Stimulation

In *Cyanobium* sp. CYA-15, La caused an initial decrease in cell density followed by rapid growth, with the maximum density approximately twofold higher than that of the control. This biphasic response is consistent with initial toxic shock, during which a fraction of the population underwent regulated cell death (RCD) [77,78], followed by a hormetic response—a dose-dependent phenomenon in which low or sublethal doses of a stressor stimulate biological performance [36]. Similar delayed hormetic responses have previously been reported for bacteria exposed to low concentrations of rare earth elements (REEs), where growth stimulation occurs during the later stages of cultivation following an initial disturbance of cellular homeostasis [79]. The small culture volume (9 mL) and high initial cell density (up to 10^5^ cells∙mL^−1^) probably contributed to the early decline of both cultures, as both factors can accelerate nutrient depletion and promote an earlier transition [80,81]. Thus, while La appears to modify growth dynamics, it did not prevent eventual culture decline.

The survival experiment supports a broader protective effect. Under control suboptimal light and temperature conditions, *Cyanobium* sp. CYA-15 and *Salileptolyngbya* sp. CYA-8 died by day 14, whereas *D. tharense* CYA-1 and *T. mysidocida* CYA-10 died by day 35. In contrast, under the same stressful conditions but in the presence of La, all four strains remained viable through the 35-day experiment and retained normal cellular morphology without visible signs of degradation. Moreover, visual comparison with cultures grown under optimal control conditions suggested brighter pigmentation and denser biofilm formation in the La-treated cultures. The physiological basis of this response remains unclear, and visual observations alone do not establish increased biomass production. Nevertheless, the consistent survival response indicates that La modified stress tolerance among taxonomically distinct strains.

### 4.2. Bioaccumulation and Localization of Lanthanum in Filamentous Cyanobacteria

Preliminary ICP-MS results demonstrated that all three filamentous cyanobacterial strains were capable of hyperaccumulating La. After 30 days of incubation, the La concentration reached 9.5–13.3 mg∙g^−1^ dry weight, representing 110- to 226-fold enrichment relative to the control. These values are comparable to those reported for *Limnospira platensis* (15.9 mg∙g^−1^ at 10 mg La∙L^−1^; 39.8 mg∙g^−1^ at 30 mg La∙L^−1^), indicating a dose-dependent accumulation process [22]. A similar relationship has been reported for *Microcystis aeruginosa*, in which La sorption capacity increased with increasing La concentration in the medium, reaching 208 mg∙g^−1^ [82], the highest value reported to date for living cyanobacteria. For comparison, the sorption capacity of lyophilized (non-living) cyanobacterial biomass ranges from 44 to 91.5 mg La∙g^−1^ dry weight across 12 different species [15]. Thus, our results demonstrate a strong accumulation capacity but should not be interpreted as definitive quantitative evidence of maximum sorption capacity because only one exposure concentration was tested with single measurement per treatment.

SEM-EDX analysis also showed that filament disruption resulted in a marked increase in the relative abundance of biogenic elements (C, N, and P) within the interaction volume, reflecting the release of intracellular material. The accumulated La was associated with material exposed after filament disruption, and was rarely detected in intact filaments. Together, these observations support the hypothesis of predominantly intracellular localization of this element. However, SEM-EDX is semiquantitative, probes a micrometer-scale interaction volume, and cannot distinguish cytosol from intracellular inclusions or cell-envelope material. Because the biomass was not washed with EDTA before analysis, a contribution from strongly surface-bound La also cannot be excluded. Future work should include fractionation protocols to quantify extracellular and intracellular fractions.

The strains differed in the magnitude and probable route of accumulation. *D. tharense* CYA-1 contained the highest absolute La concentration, whereas *Salileptolyngbya* sp. CYA-8 showed the greatest relative enrichment despite the lowest control value. The latter strain possesses a multilayered sheath that forms an amorphous extracellular polymeric substance (EPS) matrix [56], and the approximately two-fold increase in sulfur (S) concentration is compatible with enhanced production of sulfated EPS. Negatively charged carboxyl, hydroxyl, and sulfate groups play a major role in REE binding [15,16,45]. EPS may therefore facilitate capture at the cell surface before subsequent internalization, but the present data do not quantify the relative extracellular and intracellular fractions. In *D. tharense* [54] and *T. mysidocida* [57], the sheaths are thin, tightly fitting to the trichome but durable, and the lack of S accumulation in their biomass indicates that they rely on alternative, non-sulfated EPS or different defense mechanisms.

Exposure to La resulted in marked increases in the concentrations of light REEs (Ce, Pr, Nd, Sm, and Eu), indicating that uptake was not completely element-specific. The capacity of living cyanobacteria to accumulate REEs varies widely among species and individual elements. For example, in *Limnospira platensis*, reported concentrations range from approximately 0.7 mg∙g^−1^ for Tb and 2.2 mg∙g^−1^ for Yb to 9–14 mg∙g^−1^ for Sm and Nd, reaching 27–30 mg∙g^−1^ (Eu, Pr, Gd) [20,21,22,23]. However, the apparent enrichment of Gd is likely overestimated due to mass spectral interferences arising from oxide, hydroxide, and hydrate species formed by the more abundant light REEs. The formation of REE-derived oxide and hydroxide polyatomic ion species in the ICP-MS plasma is a well-recognized source of analytical interference, particularly affecting Eu, Gd, Tb, Yb, and Lu determinations [83]. This highlights a critical limitation: without interference correction, Gd data cannot be reliably interpreted. Background REEs in natural seawater (about 15 ng∙L^−1^) [13], medium components, or the La salt may also explain the concentrations of REEs detected in control biomass. Our results further demonstrate that marine benthic cyanobacteria are capable of substantial but strain-specific REE accumulation over a broad concentration range, while accurate assessment of selectivity will require interference-controlled analyses and direct mass balances.

### 4.3. Physiological Response and Adaptation Models to REEs

The accumulation of La in cyanobacterial biomass was accompanied by apparent changes in elemental homeostasis, affecting both key macronutrients (Na, K, Ca, Mg, P, S) and trace elements (Fe, Zn, Cu, Co, Ni, Cr, V, Se). The limited elemental-composition dataset precludes statistical testing and means that the observed changes, although consistent across several elements and strains, should be viewed as preliminary. They serve to generate hypotheses about strain-specific physiological responses. Although all three strains contained the essential elements required for cellular functioning under control conditions, their initial elemental profiles differed quantitatively, which may have contributed to the strain-specific responses observed during La exposure.

A common response among all three strains was a decrease in Ca concentration. This is consistent with the proposed role of La as a Ca antagonist and of calcium ion-channels blocker, and with competition between La^3+^ and Ca^2+^ for negatively charged binding sites and Ca-dependent transport systems [15,34,44]. By contrast, Na and K concentrations increased in the marine *T. mysidocida* CYA-10 and halotolerant *Salileptolyngbya* sp. CYA-8 but decreased in *D. tharense* CYA-1, indicating differences in the regulation of osmotic balance and membrane potential [84,85,86]. The increase in Mg concentration observed in all strains may represent a protective response, as Mg^2+^ functions as a cofactor for numerous enzymes and contributes to the stabilization of cellular structures under stress conditions [87,88].

The apparent decrease in phosphorus content observed in the biomass of *T. mysidocida* CYA-10 and *D. tharense* CYA-1 following La exposure could be related to the formation of phosphates by the remaining free lanthanum ions in the working medium, thereby leading to lower phosphate bioavailability. In contrast, P levels were maintained in *Salileptolyngbya* sp. CYA-8. The reference proteome of its closest available relative (*Leptolyngbya iicbica* LK) revealed the presence of proteins associated with the C-P lyase pathway (subunits PhnG, PhnH, PhnI, PhnK, PhnL) and phosphonate-transport and signaling systems (substrate-binding protein, permease PhnE, and ATP-binding protein, PhnF, PhoU), which may provide *Salileptolyngbya* sp. CYA-8 with greater flexibility in phosphorus acquisition [89,90]. These proteins were not identified in the reference proteomes used for comparison with the other two strains.

The apparent 200- to 485-fold increase in the Se signal is notable, but its magnitude may be affected by possible Gd-related mass-spectrometric interference [91]. Moreover, in the reference proteomes analyzed lack a complete selenocysteine-incorporation system, and the observed Se accumulation is unlikely to be associated with increased synthesis of selenoproteins, since cyanobacteria generally do not possess widespread Se-dependent glutathione peroxidase and selenoproteins [92]. The consistent upward trend in Se concentrations across all strains suggests a biological response and may represent a detoxification or stress-response mechanism involving sequestration into less reactive forms, which were not determined. The strain-specific changes in sulfur content observed in this study and the confirmed presence of glutathione-related enzymes (including glutathione peroxidase (GPx), glutathione reductase (GR), glutathione synthetase (GS), glutathione S-transferases (GST), and glutaredoxin (Grx)) in the reference proteomes are consistent with altered thiol-based antioxidant metabolism, although this requires biochemical confirmation.

The changes in zinc (Zn), copper (Cu), iron (Fe), manganese (Mn), molybdenum (Mo), and nickel (Ni) concentrations suggest that oxidative defense and nitrogen metabolism were reorganized differently in each strain. These metals are essential micronutrients in cyanobacteria and serve as cofactors in numerous enzymes involved in metabolic processes [76]. *Salileptolyngbya* sp. showed a 37-fold increase in Ni concentration, suggesting a potential shift in Ni-dependent metabolic processes. Thus, Ni-superoxide dismutase (sodN) was identified in the reference proteome of its closest relative, making enhanced Ni-dependent antioxidant activity a plausible hypothesis. Ni-SOD is characteristic of some marine cyanobacteria and provides an efficient mechanism for detoxifying superoxide under oxidative stress [93].

In contrast to *Salileptolyngbya* sp. CYA-8, no Ni-SOD proteins were identified in the reference proteomes used for *D. tharense* or *T. mysidocida.* The absence of Ni accumulation in these two strains under La exposure is consistent with the possibility that they rely on alternative SOD types [93,94,95], but this requires enzymatic validation. In the reference proteomes used for *D. tharense* and *T. mysidocida*, Fe-SOD and Mn-SOD, as well as cambialistic Fe/Mn-SOD that can utilize either metal cofactor, were identified. Given the marked decrease in iron (Fe) content observed following La exposure, it is plausible that Mn-dependent antioxidant enzymes become increasingly important in these strains. This interpretation is consistent with the well-documented ability of cyanobacteria to replace or supplement Fe-dependent SODs with Mn-SOD or cambialistic Fe/Mn-SOD isoforms under iron-limiting conditions [96,97].

The increase in Ni concentration in *Salileptolyngbya* sp. CYA-8 may also be associated with the optimization of nitrogen metabolism. The elevated initial boron concentration in this strain may be associated with diazotrophic activity [98]. Under lanthanum-induced stress, increased availability of Ni may supports the additional protection of nitrogenase against oxygen [99]. Ni is an essential cofactor of urease [100], potentially providing an additional nitrogen source from urea. In *T. mysidocida* and *D. tharense*, Ni decreased while Mo increased moderately. Their reference proteomes contain molybdenum-dependent nitrate reductase (Mo-NR), suggesting a different adjustment of nitrogen assimilation. Such metabolic flexibility may be advantageous under stress conditions, when phosphorus availability and energy metabolism are affected [101]. The concurrent increase in copper and zinc in *D. tharense* and *Salileptolyngbya* sp. is unlikely to reflect induction of Cu/Zn-SOD, because this enzyme class is relatively uncommon among cyanobacteria and was not identified in the reference proteomes. Instead, elevated Cu and Zn levels may be associated with broader stress-response and general repair processes.

Cobalt concentrations decreased in all strains following lanthanum exposure and may reflect an energy-saving strategy: downregulation of the energetically costly biosynthesis of pseudocobalamin [102,103], especially under Fe limitation [104].

All three cyanobacterial strains possess an extensive repertoire of iron transport and storage proteins in their corresponding reference proteomes, including FeoB, FTR1, ABC-type iron transporters, FetB, FeoA-like, ferritin, and flavodoxin as an iron-independent electron carrier. Additionally, the proteome of *Desertifilum tharense* IPPAS B-1220 contains a TonB-dependent siderophore receptor and proteins involved in siderophore biosynthesis, suggesting the potential for high-affinity iron acquisition. Despite this potential, lanthanum exposure resulted in a pronounced decrease in Fe concentration, indicating that iron homeostasis was substantially affected. The available elemental and in silico data support these hypotheses but do not demonstrate enzyme activity, cofactor incorporation, or pathway induction.

Vanadium concentrations increased in *D. tharense* and remained stable in *Salileptolyngbya* sp. and *T. mysidocida*. One possible explanation is the involvement of vanadium-dependent haloperoxidase (V-HPO) in antioxidant defense.

Elemental analysis revealed distinct strain-specific patterns of heavy metal dynamics following La exposure. *T. mysidocida* CYA-10 exhibited decreases in Pb, Cr, Ag, Ba, Sr, and Cd, suggesting a more effective regulation of intracellular heavy metal homeostasis than the other two strains. Among the three strains, *T. mysidocida* CYA-10 had the highest initial Ba (23.5 μg∙g^−1^) and Ag (20.9 μg∙g^−1^) concentrations. In cyanobacteria containing polyphosphate and carbonate granules (e.g., *Gloeomargarita lithophora* and *Cyanothece* sp.), Ba, and Ca, have been shown to accumulate within these inclusions [105]. Polyphosphate granules are recognized as important compartments for the sequestration of excess divalent and trivalent cations through electrostatic interactions and co-precipitation [101,106]. The abundant polyphosphate granules observed microscopically in *T. mysidocida* CYA-10, together with their absence in *Salileptolyngbya* sp. and only fine granulation in *D. tharense*, suggest that polyphosphate storage may contribute to the distinct metal-handling strategy of this strain. In *T. mysidocida*, La likely displaces calcium, barium, and heavy metals from these granules, as evidenced by the marked decrease in Ba (23.5→4.5 μg∙g^−1^) and the moderate decrease in Ca. This compartmentalization minimizes the cytosolic concentration of toxic La^3+^ and thus reduces oxidative stress, which may explain why *T. mysidocida* did not require massive upregulation of SOD cofactors. However, elemental colocalization within individual granules was not demonstrated, so active efflux, reduced uptake, and sequestration remain alternative or complementary explanations. The reference proteomes of all three strains contained polyphosphate kinase, but the phenotypic expression of polyphosphate granules generation appears to be strain-specific and this detoxification mechanism is likely the key resistance trait in *T. mysidocida*.

In *D. tharense* CYA-1, La exposure was accompanied by decreases in Pb, Cd, and Sr concentrations, whereas Cr, Ag, and Ba increased. In contrast, *Salileptolyngbya* sp. CYA-8 accumulated Cr, Ba, Pb, and Sr, but exhibited reduced Cd and Ag concentrations. These contrasting patterns suggest strain-specific differences in heavy metal homeostasis, although confirmatory analyses are needed. In *Salileptolyngbya* sp., the thick, multilayered EPS matrix may provide an additional passive barrier by binding incoming metal ions before they reach the cell surface, while intracellular transport systems may contribute to maintaining low concentrations of particularly toxic metals such as Cd. The EPS-based defense appears highly effective, as *Salileptolyngbya* sp. maintained normal or even increased levels of macronutrients (P, S, Mg, Mn) and showed survival under suboptimal conditions. The pronounced strain-specific responses to La and other metals are consistent with classical studies showing that even in one model cyanobacterium (*Anacystis nidulans*), toxicity and threshold concentrations for different metals (Hg, Cd, Mn, Zn) differ significantly [107,108]. Together, our findings indicate that La tolerance does not arise from a single conserved mechanism. Instead, each strain appears to combine ion regulation, extracellular binding, intracellular sequestration, and antioxidant adjustment in different proportions. Interpretation should account for the principal limitations of the study, and dose-response experiments, time-resolved uptake measurements, direct separation of extracellular and intracellular fractions, and transcriptomic or enzymatic analyses are needed to test the proposed mechanisms. Despite these limitations, the data provide a valuable first insight into the elemental rearrangements accompanying La hyperaccumulation and serve as a basis for hypothesis generation.

### 4.4. Biotechnological Potential and Strain-Specific Applications

The strain-specific responses and metal accumulation patterns observed among the three filamentous cyanobacterial strains highlight their future potential for different biotechnological applications within circular-economy approaches to REE management.

#### 4.4.1. *Desertifilum tharense* CYA-1 as a Candidate Bioindicator of REE Pollution

Among the studied strains, *D. tharense* accumulated the highest absolute La concentration (13.3 mg∙g^−1^) and exhibited the most pronounced changes in elemental homeostasis. Although the present study employed a relatively high La concentration (10 mg∙L^−1^) to elicit measurable physiological responses, this concentration served primarily as a screening dose to characterize the range and nature of stress responses. Previous studies have shown that cyanobacteria can respond to REEs at concentrations several orders of magnitude lower. For example, *Nostoc linckia* exposed to 0.001 mg∙L^−1^ La^3+^ exhibited a 4.2-fold decrease in chlorophyll a content and a 2.2-fold increase in malondialdehyde (MDA) [19]. The multiparameter response profile established for *D. tharense*—including alterations in ion homeostasis (K, Na, Ca, Fe), phosphorus and sulfur metabolism, and shifts in SOD cofactor metals—provides a set of sensitive physiological perturbation following La exposure, suggesting that it may represent a promising candidate bioindicator for REE contamination. Its broad geographic distribution [54,60,61] further supports its potential applicability across diverse environments, but its specificity and sensitivity relative to other taxa require further validation.

#### 4.4.2. *Toxifilum mysidocida* CYA-10 as a Platform for REE Recovery and Bioprospecting

Among the three strains, *T. mysidocida* demonstrated high La accumulation (11.2 mg∙g^−1^), active efflux of toxic metals, apparent intracellular sequestration of La in polyphosphate granules, and comparatively limited increases in antioxidant-metal cofactors. These features are compatible with efficient intracellular detoxification and make the strain, or its metal-handling mechanisms, of interest for REE biorecovery [109]. The presence of numerous well-defined polyphosphate granules observed microscopically, together with the marked decrease in Ba and the moderate reduction in Ca concentrations, suggests that *T. mysidocida* effectively compartmentalizes La, minimizing its cytosolic toxicity. Its ability to co-accumulate light REEs (Ce, Pr, Nd, Sm, Eu, Gd) indicates potential for mixed REE wastewater treatment. This intracellular, selective accumulation yields a relatively pure La-rich concentrate after cell lysis, making *T. mysidocida* a superior candidate for REE recovery from solutions containing mixed metal contaminants. Although the recovery requires cell disruption and acid leaching to solubilize LaPO_4_ from polyphosphate granules, the increased purity of the final product may justify the additional processing steps in a circular economy framework. Additionally, the control biomass of this strain contained notably high silver concentrations, suggesting that it may also be considered for silver recovery from solutions under controlled conditions. Because *T. mysidocida* is toxin-producing [57], its practical application would require appropriate containment and biomass management. Nevertheless, the mechanisms underlying its apparent metal tolerance, particularly those associated with polyphosphate metabolism, may provide valuable targets for the development or engineering of environmentally safe cyanobacterial strains with enhanced REE-recovery capacity for large-scale cultivation.

#### 4.4.3. *Salileptolyngbya* sp. CYA-8 as a Candidate for REE Bioremediation

*Salileptolyngbya* sp. CYA-8 exhibited the highest relative enrichment of La (226-fold), maintained P homeostasis, and formed an EPS-rich biofilm. These characteristics, together with successful cultivation on microcarriers in a suspended-solid-phase photobioreactor [26], make it an attractive candidate for attached-growth photobioreactors designed for bioremediation of polymetallic wastewaters. The approximately twofold increase in sulfur concentration following La exposure is consistent with enhanced production of sulfated EPS components—a well-known metal-induced protective response [46,110,111]. The EPS-mediated non-selective binding of La^3+^ and multiple metals (Cr, Ba, Pb, Sr), followed by intracellular transport and Ni-SOD activation, represents a potential dual-phase mechanism: surface adsorption enabling easy desorption, combined with intracellular accumulation for higher capacity. Unlike the other two strains, the strain maintained stable phosphorus concentrations under La exposure, which is consistent with the presence of genes associated with phosphonate and phosphite utilization (C–P lyase pathway) identified in the reference proteome of its closest phylogenetic relative. This metabolic flexibility could provide an advantage during large-scale cultivation in nutrient-variable wastewaters. Repeated-cycle performance, extracellular/intracellular partitioning, non-selectivity, and desorption efficiency must therefore be measured before the feasibility of mild chelation and biomass reuse for industrial applications can be established [20,21,48,112].

In summary, *D. tharense* CYA-1, *T. mysidocida* CYA-10 and *Salileptolyngbya* sp. IBSS-CYA-8 from Black Sea periphyton represent potentially complementary resources for REE monitoring, recovery, and remediation, respectively. The next priorities are to determine environmentally relevant response thresholds, optimize cultivation and desorption conditions, quantify metal mass balances, and verify the molecular mechanisms responsible for strain-specific accumulation and tolerance.

## 5. Conclusions

This study provides initial evidence that marine benthic cyanobacteria exhibit strain-specific responses to lanthanum. At sublethal concentrations, La may act as a potent biological modulator, inducing a delayed hormetic response that stimulates the growth of *Cyanobium* sp. CYA-15. Lanthanum also provided long-term protection against light and temperature stress in all strains studied. Simultaneously, the cyanobacteria exhibited hyperaccumulation of lanthanum and co-accumulation of several light rare earth elements, reaching concentrations comparable to those reported for known REE-hyperaccumulating cyanobacteria. SEM-EDX analysis provided strong evidence of predominantly intracellular La localization, confirming that bioaccumulation, rather than passive biosorption, is the primarily mechanism. Lanthanum exposure was associated with apparent strain-specific changes in elemental homeostasis. While several responses were consistently observed across strains, distinct adaptive patterns were also noted. The observed trends, together with the SEM-EDX and phenotypic evidence, support the hypothesis of three different protective strategies. *T. mysidocida* likely employs metal efflux and polyphosphate granule sequestration; *Salileptolyngbya* sp. may upregulate sulfated EPS production and Ni-SOD synthesis; *D. tharense* may rely on Mn-SOD. These strain-specific characteristics also highlight different biotechnological opportunities. *D. tharense* represents a promising candidate bioindicator for REE pollution, *T. mysidocida* offers a platform for REE recovery, and *Salileptolyngbya* sp., which exhibits EPS-mediated REE capture and has demonstrated scalability in photobioreactors, is a promising candidate for REE and heavy metal bioremediation. The strain specificity of these responses underscores that the adaptive response to REE exposure is unique to each strain. These findings deepen our fundamental understanding of REE–cyanobacteria interactions and open new avenues for bioremediation, bioindication, and REE recovery aligned with circular economy principles, but only under carefully controlled and validated conditions. Future work should scale up cultivation, optimize desorption, include replicated analyses, and explore the genetic basis of strain-specific metal tolerance.

## Data Availability

All data used in this study are available upon request from the corresponding author. 16S rRNA sequence of strain *Salileptolyngbya* sp. IBSS-CYA-8 (648 bp linear DNA) have been submitted to GenBank (URL: https://www.ncbi.nlm.nih.gov/nuccore/PV984064, accessed on 11 December 2025).

## Supplementary Materials

The following supporting information can be downloaded at https://www.mdpi.com/article/10.3390/biology15171551/s1, Table S1: Concentration (wt%) of the main elements in intact and destroyed filaments compared to the background; Table S2. Concentration of elements in the biomass of the studied cyanobacterial strains after 30 days of cultivation in control medium (C) and La-supplemented medium (+La).
