# Supplementary material for: Marine Benthic Cyanobacteria as Potential Bioresources for Rare Earth Element Management: Insights from Lanthanum Bioaccumulation and Strain-Specific Physiological Responses
Source: Biology (Basel). 2026 Sep 4;15(17):1551. doi: 10.3390/biology15171551 (PMC13565681; doi:10.3390/biology15171551)
Supplement: Supplementary file 1 [file biology-15-01551-s001.zip › biology-4512959-supplementary.pdf]

Supplementary Material

Marine Benthic Cyanobacteria as Potential Bioresources for Rare Earth Element Management: Insights from Lanthanum Bioaccumulation and Strain-Specific Physiological Responses

Ekaterina Sergeevna Miroshnichenko [1], Anastasiia Andreevna Blaginina [1], Daria Sergeevna Balycheva [1,*], Vyacheslav Nikolaevich Lishaev [1], Sergey Victorovich Kapranov [1], Mikhail Vitalievich Simokon [2], Svetlana Nikolaevna Zheleznova [1] and Vitaliy Ivanovich Ryabushko [1]

Table S1. Concentration (wt %) of the main elements in intact and destroyed filaments compared to the background.

**Element, *wt* %**	***D. tharense* CYA-1**		***T. mysidocida* CYA-10**		***Salileptolyngbya* sp. CYA-8**		**Background**
	Intact	Destroyed	Intact	Destroyed	Intact	Destroyed	
**O**	42.85<br>(41.28 – 44.85)<br>*n* = 30	44.2<br>(40.3 – 46.25)<br>*n* = 33	43.6<br>(40.6 – 45.6)<br>*n* = 31	41.3<br>(40.1 – 42.5)<br>*n* = 31	44<br>(40.9 – 45.48)<br>*n* = 36	40.45<br>(36.78 – 43.43)<br>*n* = 30	47.25<br>(46.38 – 47.7)<br>*n* = 90
**Si**	25.7<br>(22.38 – 29.63)<br>*n* = 30	24.7<br>(16.75–26.75)<br>*n* = 33	26.3<br>(23.9 – 28.2)<br>*n* = 31	20.2<br>(18.2 – 22.2)<br>*n* = 31	28.75<br>(24.1 – 30)<br>*n* = 36	19.9<br>(17.65 – 25.4)<br>*n* = 30	30.3<br>(28.35 – 30.7)<br>*n* = 90
**C**	17.5<br>(10.78 – 22.18)<br>*n* = 30	16.2<br>(12 – 28.3)<br>*n* = 33	16.1<br>(11.6 – 21.9)<br>*n* = 31	22.9<br>(21.2 – 26.1)<br>*n* = 31	13.2<br>(8.2 – 20.55)<br>*n* = 36	24.25<br>(14.4 – 30.58)<br>*n* = 30	6.9<br>(5.9 – 10.1)<br>*n* = 90
**Na**	6.3<br>(5.7 – 6.83)<br>*n* = 30	5.7<br>(3.8 – 6.4)<br>*n* = 33	6.3<br>(5.7 – 6.8)<br>*n* = 31	4.7<br>(4.2 – 5.1)<br>*n* = 31	6.8<br>(6.03 – 7.3)<br>*n* = 36	4.65<br>(3.98 – 6.2)<br>*n* = 30	7.5<br>(7.1 – 7.6)<br>*n* = 90
**Ca**	4.25<br>(3.7 – 5)<br>*n* = 30	4.3<br>(3.9 – 4.65)<br>*n* = 33	4.4<br>(4 – 4.6)<br>*n* = 31	3.8<br>(3.3 – 4.1)<br>*n* = 31	4.95<br>(4.2 – 5.2)<br>*n* = 36	4.1<br>(3.4 – 4.83)<br>*n* = 30	5<br>(4.6 – 5.1)<br>*n* = 90
**Mg**	2.1<br>(1.9 – 2.33)<br>*n* = 30	3.1<br>(2.8– 4.2)<br>*n* = 33	2.3<br>(2.1 – 2.4)<br>*n* = 31	3.1<br>(2.7 – 4.1)<br>*n* = 31	2.2<br>(1.93 – 2.30)<br>*n* = 36	2.35<br>(2.08 – 3.33)<br>*n* = 30	2.4<br>(2.3 – 2.4)<br>*n* = 90
**K**	0.3<br>(0.2 – 0.3)<br>*n* = 30	0.3<br>(0.2 – 0.3)<br>*n* = 33	0.3<br>(0.2 – 0.3)<br>*n* = 31	0.2<br>(0.2 – 0.3)<br>*n* = 31	0.3<br>(0.3 – 0.3)<br>*n* = 36	0.2<br>(0.2 – 0.3)<br>*n* = 30	0.3<br>(0.3 – 0.3)<br>*n* = 90
**N**	1.9<br>(1.6 – 2.4)<br>*n* = 13	3.5<br>(1.85 – 4.05)<br>*n* = 10	1.3<br>(1.15 – 2.6)<br>*n* = 17	1.95<br>(1.7 – 2.65)<br>*n* = 26	2.1<br>(1.55 – 2.65)<br>*n* = 9	2.95<br>(2.18 – 4.48)<br>*n* = 14	*nd*
**P**	*nd*	0.4<br>(0.3 – 0.73)<br>*n* = 18	0.1<br>*n* = 1	0.3<br>(0.2 – 0.5)<br>*n* = 24	*nd*	0.55<br>(0.4 – 0.88)<br>*n* = 20	*nd*
**S**	0.1<br>(0.1 – 0.18)<br>*n* = 12	0.2<br>(0.1 – 0.2)<br>*n* = 20	0.1<br>(0.1 – 0.1)<br>*n* = 12	0.2<br>(0.2 – 0.2)<br>*n* = 28	0.1<br>(0.1 – 0.2)<br>*n* = 8	0.2<br>(0.2 – 0.3)<br>*n* = 23	*nd*
**Cl**	*nd*	0.1<br>*n* = 1	0.1<br>(0.1 – 0.1)<br>*n* = 8	0.1<br>(0.1 – 0.1)<br>*n* = 25	0.2<br>(0.1 – 0.2)<br>*n* = 8	0.25<br>(0.2 – 0.3)<br>*n* = 22	*nd*
**La**	0.05<br>*n* = 1	0.35<br>(0.2 – 0.7)<br>*n* = 20	0.2<br>*n* = 1	0.3<br>(0.2 – 0.5)<br>*n* = 19	0.2<br>(0.08 – 0.2)<br>*n* = 5	0.5<br>(0.3 – 0.8)<br>*n* = 21	*nd*
**La/C**	0.0029	0.0216	0.0124	0.0131	0.0152	0.0206	
**La/(C+N+P)**	0.0026	0.0174	0.0114	0.0119	0.0131	0.0180	

Notes: *Values are medians (25th – 75th percentiles), n = number of detections, nd = not detected.*

Table S2. Concentration of elements in the biomass of the studied cyanobacterial strains after 30 days of cultivation in control medium (C) and La-supplemented medium (+ La).

**Element, µg/g dry weight***	***Salileptolyngbya* sp. CYA-8**			***T. mysidocida* CYA-10**			***D. tharense* CYA-1**		
	**+ La**	**C**	*+ La / C*	**+ La**	**C**	*+ La / C*	**+ La**	**C**	*+ La / C*
Rare earth elements									
**Lanthanum (La)**	**9516.28**	42.02	*226.46*	**11228.84**	83.792	*134.00*	**13320.70**	120.669	*110.39*
**Cerium (Ce)**	2.142	0.465	*4.61*	2.542	0.169	*15.04*	2.389	0.082	*29.13*
**Praseodymium (Pr)**	0.063	0.007	*9.04*	0.080	0.011	*7.03*	0.081	0.004	*20.25*
**Neodymium (Nd)**	0.232	0.022	*10.44*	0.307	0.040	*7.70*	0.246	0.014	*17.57*
**Samarium (Sm)**	0.158	0.005	*34.70*	0.157	0.010	*15.70*	0.192	0.003	*64.00*
**Europium (Eu)**	0.043	0.003	*15.28*	0.046	0.010	*4.60*	0.058	0.003	*19.33*
**Gadolinium (Gd)***	1.538	0.015	*102.52*	2.141	0.018	*118.94*	1.980	0.013	*152.31*
**Dysprosium (Dy)**	0.006	0.004	*1.50*	0.010	0.009	*1.11*	0.008	0.003	*2.66*
**Holmium (Ho)**	0.005	0.002	*2.50*	0.004	0.003	*1.33*	0.004	0.001	*4.00*
**Erbium (Er)**	0.027	0.112	*0.24*	0.114	0.016	*7.13*	0.037	0.006	*6.16*
**Thulium (Tm)**	0.001	0.001	*1.00*	0.001	0.002	*0.50*	0.001	0.0005	*2.00*
**Ytterbium (Yb)**	0.011	0.005	*2.20*	0.014	0.011	*1.27*	0.012	0.003	*4.00*
**Lutetium (Lu)**	0.002	0.002	*1.00*	0.002	0.002	*1.00*	0.001	0.0005	*2.00*
Macronutrients									
**Sodium (Na)**	13.39	3.28	*4.08*	33.32	18.20	*1.83*	15.84	20.93	*0.76*
**Potassium (K)**	6.94	4.05	*1.71*	6.30	4.13	*1.53*	3.08	7.38	*0.42*
**Calcium (Ca)**	1.07	1.46	*0.73*	1.28	3.36	*0.38*	1.08	3.68	*0.29*
**Magnesium (Mg)**	55.98	53.01	*1.06*	57.88	36.36	*1.59*	60.28	28.00	*2.15*
**Phosphorus (P)**	10.11	10.26	*0.99*	5.94	15.12	*0.39*	5.86	17.24	*0.34*
**Sulfur (S)**	1.93	0.91	*2.12*	2.47	2.33	*1.06*	1.43	2.02	*0.71*
Trace elements									
**Selenium (Se)***	502.70	1.04	*483.37*	762.06	2.66	*286.49*	728.88	3.64	*200.24*
**Iron (Fe)**	567.19	1147.92	*0.49*	1620.83	2035.70	*0.80*	689.80	1686.02	*0.41*
**Manganese (Mn)**	62.42	65.09	*0.96*	57.57	84.65	*0.68*	84.96	61.81	*1.37*
**Copper (Cu)**	36.29	14.24	*2.55*	21.49	21.82	*0.98*	14.98	9.34	*1.60*
**Zinc (Zn)**	141.86	62.15	*2.28*	91.56	106.57	*0.86*	215.40	78.49	*2.74*
**Cobalt (Co)**	3.29	6.50	*0.51*	1.72	2.45	*0.70*	2.45	3.86	*0.63*
**Molybdenum (Mo)**	3490.18	4276.72	*0.82*	7649.89	4758.21	*1.61*	5985.60	2712.55	*2.21*
**Nickel (Ni)**	105.89	2.87	*36.90*	1.51	4.95	*0.31*	1.79	2.06	*0.87*
**Vanadium (V)**	1.08	0.81	*1.33*	1.48	1.40	*1.06*	1.20	0.50	*2.4*
**Boron (B)**	1454.40	1116.00	*1.30*	680.76	541.55	*1.26*	533.72	190.46	*2.8*
Heavy metals									
**Chromium (Cr)**	19.44	3.28	*5.93*	1.50	5.12	*0.29*	4.91	2.33	*2.11*
**Strontium (Sr)**	172.64	133.75	*1.29*	209.15	297.31	*0.70*	189.32	346.41	*0.55*
**Silver (Ag)**	2.06	3.23	*0.64*	8.90	20.87	*0.43*	4.21	3.80	*1.11*
**Cadmium (Cd)**	0.07	1.79	*0.04*	1.27	1.25	*1.01*	0.16	0.88	*0.18*
**Barium (Ba)**	11.32	3.48	*3.25*	12.35	23.47	*0.53*	37.08	5.26	*7.05*
**Lead (Pb)**	3.97	1.50	*2.65*	0.32	4.14	*0.08*	0.23	2.04	*0.11*

* Data are from single measurements (n = 1) due to limited biomass; therefore, no standard deviations or statistical significance are provided. Fold-changes (experimental/control) are indicative and should be interpreted with caution. Values for Gd and Se may be affected by mass-spectral interferences from La.
